# Anatomical Outcomes of Traumatic Macular Hole Surgery: Role of OCT Parameters and ILM Flap Techniques

**DOI:** 10.3390/jcm15156040

**Published:** 2026-08-03

**Authors:** Sehnaz Ozcaliskan, Merve Ozbek, Anil Korkmaz, Murat Arici

**Affiliations:** Ophthalmology Department, Beyoglu Eye Training and Research Hospital, Istanbul 34421, Turkey; drmerveyalcin@gmail.com (M.O.); anilkorkmaz@yahoo.com (A.K.); mrt_arici@hotmail.com (M.A.)

**Keywords:** traumatic macular hole, pars plana vitrectomy, internal limiting membrane flap, optical coherence tomography

## Abstract

**Background**: To evaluate anatomical and functional outcomes following pars plana vitrectomy (PPV) for traumatic macular hole (TMH), investigate optical coherence tomography (OCT)-based prognostic factors, and compare outcomes between flap-based and non-flap internal limiting membrane (ILM) techniques. **Methods**: This retrospective consecutive case series included 33 eyes of 33 patients undergoing PPV for TMH between January 2017 and June 2024 at a tertiary referral center. Preoperative spectral-domain OCT parameters, including minimum linear diameter (MLD), basal diameter (BD), hole height (HH), intraretinal cysts, subretinal fluid, epiretinal membrane, and ellipsoid zone disruption, were evaluated. Anatomical closure and postoperative best-corrected visual acuity (BCVA) outcomes were analyzed. **Results**: The mean patient age was 28.7 ± 15.0 years, and blunt trauma accounted for 87.9% of cases. Median trauma-to-surgery interval was 10 weeks (IQR: 4–24 weeks). Mean BCVA improved significantly from 1.34 ± 0.54 logMAR preoperatively to 0.95 ± 0.59 logMAR postoperatively (*p* = 0.004). Single-surgery anatomical closure was achieved in 28 of 33 eyes (84.8%). Larger MLD and BD were significantly associated with poorer final BCVA, whereas greater HH was associated with successful anatomical closure. Flap-assisted techniques achieved higher closure rates compared with conventional ILM peeling (95.8% vs. 55.6%, *p* = 0.013). **Conclusions**: PPV for TMH was associated with favorable anatomical and functional outcomes. Larger hole dimensions were associated with poorer visual prognosis, whereas greater HH was associated with anatomical closure. Flap-assisted ILM techniques may improve anatomical success in TMH surgery.

## 1. Introduction

Traumatic macular hole (TMH) is an uncommon but vision-threatening complication of ocular trauma, most frequently occurring after blunt injury. In contrast to idiopathic macular holes, TMH typically affects younger patients and may present with immediate or delayed visual loss [1,2]. Although spontaneous closure can occur, particularly in smaller holes and younger patients, pars plana vitrectomy (PPV) is often required in persistent, large, or visually significant cases [3,4].

Advances in optical coherence tomography (OCT) imaging have improved the characterization of TMH morphology and have enabled detailed assessment of structural prognostic factors [4,5]. Previous studies have suggested that OCT-based parameters, including minimum linear diameter (MLD), basal diameter (BD), hole height (HH), and ellipsoid zone integrity, may influence anatomical and functional outcomes [6,7]. Larger TMHs, particularly those with an MLD exceeding approximately 500 µm, have been associated with lower closure rates after conventional ILM peeling, prompting increasing use of flap-based techniques in large or complex cases [8]. Nevertheless, the optimal OCT-based criteria for predicting anatomical success and selecting the most appropriate surgical technique remain incompletely established.

Surgical management of TMH has also evolved with the use of internal limiting membrane (ILM) flap techniques, including inverted and free ILM flaps. Although these approaches have shown promising results, especially in large or complex macular holes, evidence comparing flap-based techniques with conventional ILM peeling in TMHs remains limited [9].

Therefore, the aim of this study was to evaluate anatomical and visual outcomes following PPV for TMH. We also sought to investigate preoperative OCT-based predictors of surgical outcomes and to compare anatomical success between flap-based and non-flap ILM techniques.

## 2. Materials and Methods

This single-center retrospective consecutive case series was conducted at the vitreoretinal surgery unit of a tertiary referral hospital. Medical records of patients diagnosed with TMH between January 2017 and June 2024 were retrospectively reviewed. The study adhered to the tenets of the Declaration of Helsinki and was approved by the local Institutional Review Board (Approval No: 8/3; Date: 1 August 2024).

Inclusion criteria included eyes with a history of ocular trauma associated with acute visual loss and a diagnosis of full-thickness TMH confirmed by clinical examination and spectral-domain OCT. Only eyes with a minimum postoperative follow-up duration of 6 months were included.

Eyes with macular hole associated with macula-involving rhegmatogenous retinal detachment were excluded. Additional exclusion criteria included idiopathic or myopic macular holes, previous vitreoretinal surgery, coexisting retinal disorders that could affect visual acuity or anatomical outcomes (including advanced diabetic retinopathy or age-related macular degeneration), and inadequate OCT image quality due to media opacity (e.g., traumatic cataract or corneal opacity), poor fixation, or other conditions precluding reliable assessment of macular hole morphology.

Demographic and clinical data were collected from electronic medical records, including age, sex, trauma type, affected eye, lens status, associated ocular findings, interval between trauma and surgery, preoperative and final best-corrected visual acuity (BCVA), surgical technique, tamponade agent, and combined phacoemulsification. BCVA measurements were converted to logarithm of the minimum angle of resolution (logMAR) units for statistical analysis.

Because no universally accepted protocol exists regarding the optimal timing of surgery for TMHs, patients were initially followed with serial OCT examinations to assess the potential for spontaneous closure. Continued observation was preferred in eyes demonstrating progressive reduction in hole size or signs of spontaneous closure, whereas surgery was recommended for persistent full-thickness TMHs without evidence of spontaneous improvement, progressive enlargement on serial OCT, or persistent visual impairment. Therefore, the timing of surgery was individualized according to the clinical course and OCT findings rather than a predetermined observation period. No age-specific protocol was used, and treatment decisions were based on serial OCT findings and the overall clinical course.

All surgeries were performed using a standard 23- or 25-gauge three-port PPV technique. Posterior vitreous detachment was induced when absent, with the aid of triamcinolone acetonide for vitreous visualization. Following core vitrectomy, peripheral vitreous shaving was performed circumferentially. The ILM was stained using brilliant blue G (BBG) dye to facilitate visualization. Given the retrospective design of the study, no predefined protocol was used for selecting the ILM management technique. The choice between standard ILM peeling, inverted ILM flap, and free ILM flap techniques was left to the operating surgeon’s intraoperative judgment based on individual case characteristics. Peripheral retinal breaks, retinal dialysis, or localized retinal detachment were treated with laser photocoagulation when present. Fluid–air exchange was subsequently performed, followed by tamponade using air, 20% sulfur hexafluoride (SF_6_), or 14% perfluoropropane (C_3_F_8_). All patients were instructed to maintain a prone position for at least one week postoperatively.

Preoperative SD-OCT imaging was performed using the Heidelberg Spectralis OCT system (Heidelberg Engineering, Heidelberg, Germany). All scans were acquired under standardized conditions by an experienced technician. Quantitative OCT measurements were evaluated by an experienced retinal specialist masked to clinical outcomes (O.A). The following OCT parameters were recorded: MLD, BD, HH, presence of intraretinal cysts, subretinal fluid, epiretinal membrane, and ellipsoid zone disruption.

The primary outcome measure was final postoperative BCVA at the last follow-up visit, expressed in logMAR units. Secondary outcome measures included anatomical success, defined as complete closure of the macular hole on OCT, and OCT-based factors associated with anatomical and functional outcomes. Associations between trauma-to-surgery interval and surgical outcomes were also evaluated. The relationship between trauma-to-surgery interval and both final BCVA and anatomical closure was evaluated using correlation and subgroup analyses, respectively.

### Statistical Analysis

All statistical analyses were performed using IBM SPSS Statistics version 25.0 (IBM Corp., Armonk, NY, USA). The normality of continuous variables was assessed using the Shapiro–Wilk test. Normally distributed continuous variables were expressed as mean ± standard deviation (SD), whereas non-normally distributed variables were presented as median and interquartile range (IQR). Categorical variables were expressed as number and percentage. Comparisons between groups were performed using the independent samples t-test for normally distributed continuous variables and the Mann–Whitney U-test for non-normally distributed variables. Categorical variables were compared using the chi-square test or Fisher’s exact test, as appropriate. Correlations between OCT parameters and final BCVA were evaluated using Pearson or Spearman correlation analysis according to data distribution. Associations between trauma-to-surgery interval and anatomical or functional outcomes were also evaluated. Given the limited sample size, multivariable analyses were not performed in order to avoid model overfitting. A *p*-value < 0.05 was considered statistically significant.

## 3. Results

The study included 33 eyes of 33 patients. The mean age was 28.7 ± 15.0 years, and most patients were male (93.9%). Blunt trauma was the predominant injury mechanism, observed in 29 eyes (87.9%), while penetrating trauma was present in 4 eyes (12.1%). The median interval between trauma and surgery was 10 weeks (4–24 weeks). The mean follow-up duration was 29.0 ± 27.2 months. Associated ocular findings were present in 22 eyes (66.7%). Choroidal rupture was the most common associated finding, followed by Berlin edema, mild vitreous hemorrhage, and subretinal hemorrhage (Table 1). No patient had secondary glaucoma or lens subluxation requiring surgical management at the time of TMH surgery. Combined phacovitrectomy was performed in six eyes because of traumatic cataract, and one additional patient underwent cataract surgery during follow-up because of cataract progression.

The mean MLD was 597.6 ± 282.5 µm, the mean BD was 1266.0 ± 459.2 µm, and the mean HH was 389.6 ± 130.1 µm. Preoperative OCT showed intraretinal cysts in 21 eyes (63.6%), subretinal fluid in 12 eyes (36.4%), epiretinal membrane in 11 eyes (33.3%), and ellipsoid zone disruption in 27 eyes (81.8%). BCVA improved significantly from 1.34 ± 0.54 logMAR preoperatively to 0.95 ± 0.59 logMAR at the final visit (*p* = 0.004).

Regarding surgical techniques, flap-based procedures were performed in 24 eyes (72.7%), including free ILM flap in 14 eyes and inverted ILM flap in 10 eyes. Conventional ILM peeling was performed in 9 eyes (27.3%). Air, SF_6_, and C_3_F_8_ were used for intraocular tamponade in 5 (15.2%), 15 (45.5%), and 13 (39.4%) eyes, respectively. Among eyes treated with flap-based techniques, air, SF_6_, and C_3_F_8_ were used in 20.0%, 48.0%, and 32.0% of cases, respectively, whereas the corresponding proportions in the non-flap group were 0%, 37.5%, and 62.5%. The distribution of tamponade agents did not differ significantly between the flap and non-flap groups (Fisher–Freeman–Halton exact test, *p* = 0.253).

Single-surgery anatomical closure was achieved in 28 of 33 eyes (84.8%). Eyes achieving anatomical closure had significantly greater HH than eyes without closure, with mean values of 417.1 ± 128.0 µm and 265.7 ± 24.3 µm, respectively (*p* = 0.008). In contrast, MLD and BD were not significantly associated with anatomical closure in the present cohort. Despite the absence of statistical significance, both parameters were numerically greater in eyes without anatomical closure. This finding should be interpreted cautiously, as the limited number of non-closure cases likely reduced the statistical power to detect a significant association (Table 2).

Larger MLD and BD were significantly correlated with worse final BCVA, indicating poorer functional outcomes with increasing macular hole size (r = 0.533, *p* = 0.001 and r = 0.424, *p* = 0.014, respectively). HH was not significantly correlated with final BCVA (*p* = 0.371). Preoperative BCVA showed a significant positive correlation with final BCVA (r = 0.407, *p* = 0.006). No significant associations were observed between final BCVA and ellipsoid zone disruption, intraretinal cysts, subretinal fluid, epiretinal membrane, or anatomical closure (all *p* > 0.05).

**Figure 1:**
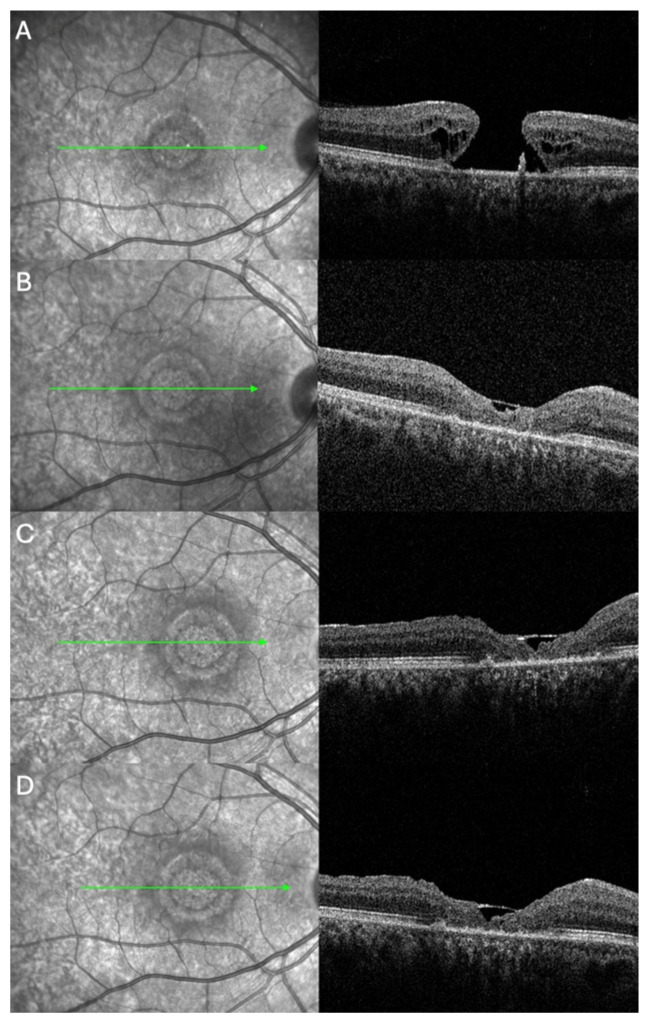
Sequential OCT images of a 41-year-old male with a chronic traumatic macular hole secondary to ocular trauma 25 years earlier. The patient underwent pars plana vitrectomy with the inverted ILM flap technique. (**A**) Preoperative image showing a full-thickness macular hole. (**B**–**D**) Postoperative months 1, 3, and 6 demonstrating successful anatomical closure and progressive foveal remodeling. Best-corrected visual acuity improved from counting fingers at 1 m preoperatively to 0.05 at month 6.

**Figure 2:**
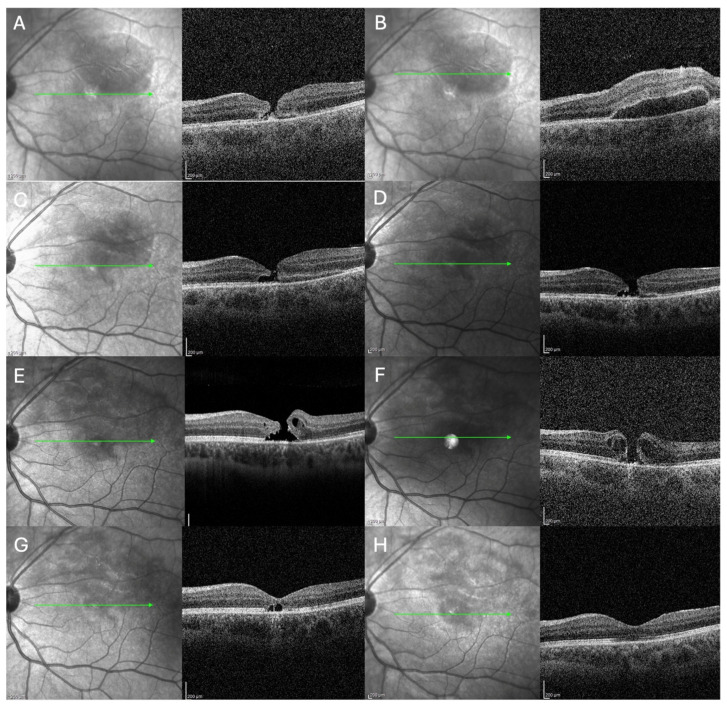
Serial OCT images of a 25-year-old male who developed a traumatic macular hole following blunt ocular trauma caused by a ball injury. (**A**,**B**) Two different OCT sections obtained on day 1 after trauma demonstrating an early full-thickness traumatic macular hole; localized subretinal fluid is evident in panel (**B**) (BCVA: 0.2). (**C**,**D**) OCT images obtained at weeks 1 and 2 showing persistence of the macular hole without spontaneous closure. (**E**,**F**) OCT images at months 1 and 2 demonstrating progressive enlargement of the hole and increasing elevation of the hole edges, prompting surgical intervention. The patient subsequently underwent pars plana vitrectomy with a free ILM flap technique. (**G**,**H**) OCT images at postoperative months 1 and 2 showing successful anatomical closure with restoration of the foveal contour. Best-corrected visual acuity improved from 0.2 preoperatively to 0.8 postoperatively.

**Figure 3:**
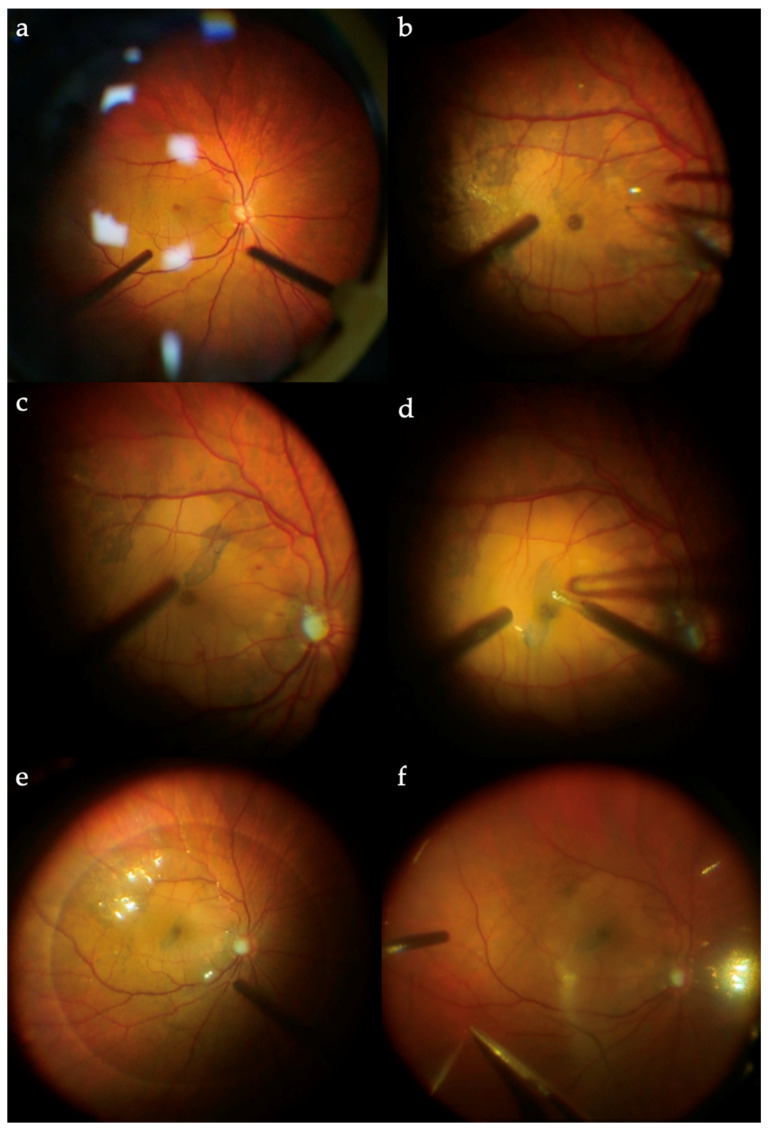
Step-by-step surgical sequence of the free internal limiting membrane (ILM) flap technique for macular hole closure. (**a**) Fundus view highlighting the macular hole. (**b**) Staining with Brilliant Blue dye demonstrating negative staining due to the presence of an epiretinal membrane (ERM). (**c**) Creation of the ILM flap through peeling. (**d**) Placement of the free ILM flap directly over the macular hole. (**e**) Confirmation of macular hole coverage and closure under perfluorocarbon liquid (PFCL). (**f**) Completion of fluid–air exchange and thorough fluid aspiration, securing the free flap in position.

**Figure 4:**
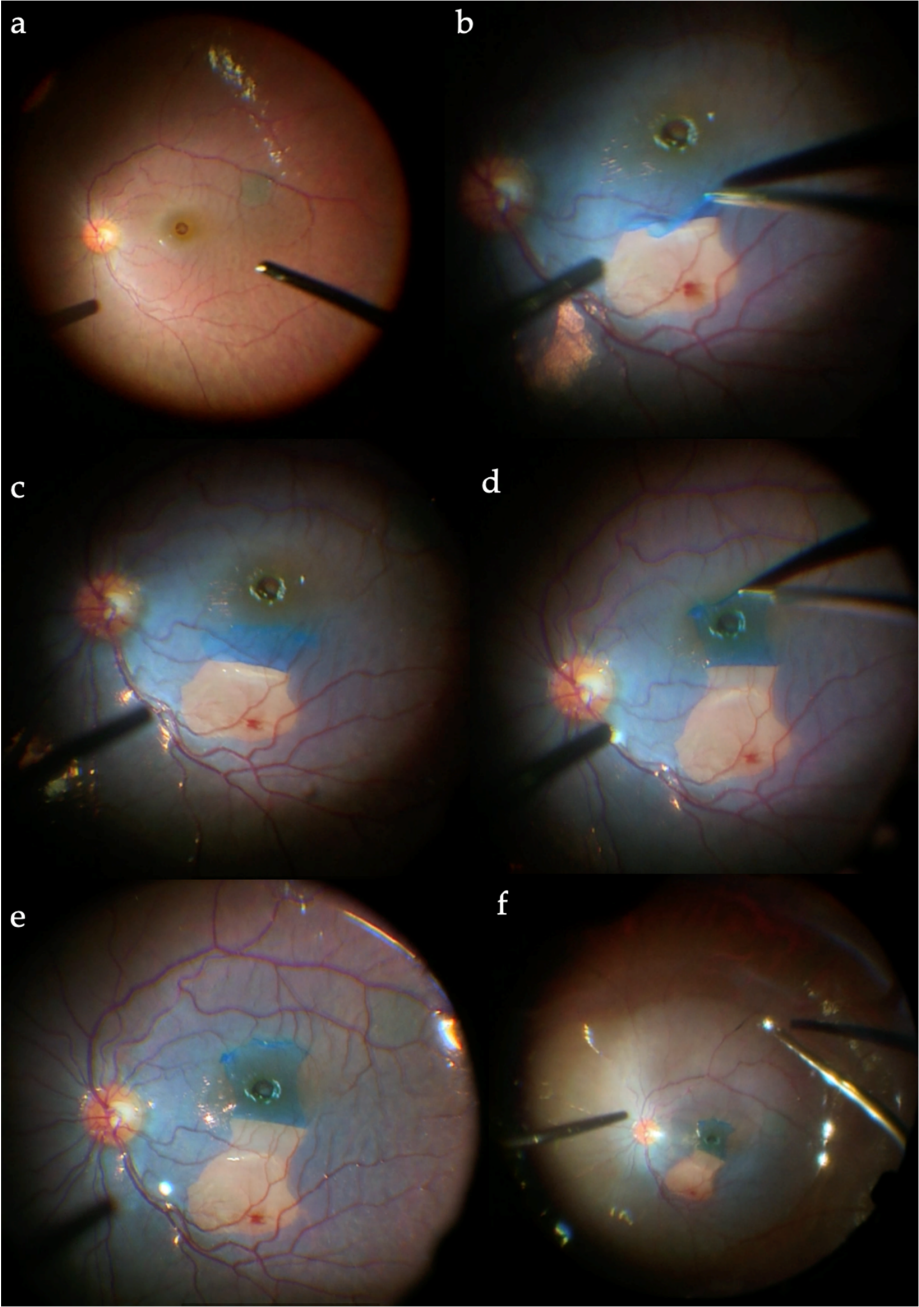
Intraoperative sequential images demonstrating the superior inverted internal limiting membrane (ILM) flap technique for macular hole repair. (**a**) Preoperative view showing a full-thickness macular hole. (**b**) Harvest of an ILM flap from the superior aspect of the hole, keeping its inferior attachment intact. (**c**) Inversion and stabilization of the flap onto the retina using perfluorocarbon liquid (PFCL). (**d**) Positioning of the flap to cover the macular hole using intraocular forceps. (**e**) Realignment and adjustment of the flap edges directly over the hole. (**f**) Complete fluid–air exchange and fluid aspiration, securing the flap in position over the macula.

Anatomical closure was achieved in 23 of 24 eyes (95.8%) in the flap group compared with 5 of 9 eyes (55.6%) in the non-flap group, demonstrating significantly higher anatomical success rates with flap-assisted techniques (Fisher’s exact test, *p* = 0.013) (Table 3). Figure 1 illustrates a representative case of chronic TMH successfully treated with the inverted ILM flap technique, demonstrating progressive foveal remodeling after surgery. Five eyes did not achieve anatomical closure after the initial surgery. Two eyes underwent revision surgery with a free ILM flap, whereas three eyes received an amniotic membrane graft. Successful anatomical closure was achieved in all five eyes after the second procedure, with no subsequent recurrence during follow-up. No significant association was observed between trauma-to-surgery interval and final BCVA or anatomical closure (all *p* > 0.05). Figure 2 demonstrates the natural course of a TMH that persisted and progressively enlarged during the observation period before surgical intervention, followed by successful anatomical closure after free ILM flap surgery. Intraoperative surgical steps for TMH repair using the superior inverted ILM flap technique and the free ILM flap technique are illustrated in Figure 3 and Figure 4, respectively. Baseline OCT parameters and preoperative BCVA were comparable between flap and non-flap groups, including MLD, BD, HH, and age (all *p* > 0.05).

## 4. Discussion

In the present study, we evaluated anatomical and functional outcomes following PPV for TMH and investigated OCT-based prognostic factors associated with surgical success. Significant postoperative visual improvement and a high rate of anatomical closure were observed. Larger macular hole dimensions, particularly MLD and BD, were associated with poorer final visual acuity, whereas greater HH was associated with successful anatomical closure. In addition, flap-based surgical techniques demonstrated higher anatomical closure rates compared with conventional ILM peeling techniques.

Single-surgery anatomical closure was achieved in 84.8% of eyes in the present cohort. This finding is generally consistent with previously reported closure rates following vitrectomy for TMH, which are commonly reported between 80% and 95%. Variability in closure rates among studies may be related to differences in patient selection, macular hole size, chronicity, surgical timing, and surgical technique [4,10]. In our cohort, many patients were referred to our tertiary center after an initial observation period for possible spontaneous closure, which may partially explain the inclusion of relatively complex or delayed cases. Nevertheless, favorable anatomical outcomes were achieved in the majority of eyes, particularly in those treated with flap-assisted techniques.

Visual acuity improved significantly after surgery; however, the degree of functional recovery varied substantially among patients. This observation is in agreement with previous studies demonstrating that anatomical closure does not always translate into complete visual recovery in TMHs [6,11,12]. The extent of visual improvement is likely influenced not only by successful hole closure, but also by associated photoreceptor damage, retinal pigment epithelium alterations, and trauma-related retinal injury. Accordingly, TMHs should be considered structurally and biologically distinct from idiopathic macular holes.

Preoperative BCVA was significantly associated with postoperative visual performance, indicating that eyes with poorer baseline vision generally achieved lower final visual acuity. This observation aligns with the prior TMH literature identifying initial visual status as one of the strongest determinants of functional recovery after surgery [6,7,11]. Conversely, the interval between trauma and surgical intervention did not demonstrate a significant relationship with either anatomical closure or final BCVA. Comparable results were described by Ghoraba et al., who similarly reported no meaningful effect of surgical timing on closure configuration or visual outcome [13]. The absence of a significant association in our series may reflect the retrospective nature of the study, variability in trauma severity, delayed referral patterns, and the relatively limited cohort size.

Morphological OCT characteristics appeared to have a substantial influence on functional prognosis. In particular, increased MLD and BD were both associated with poorer postoperative BCVA. Previous studies have likewise shown that larger TMHs tend to yield less favorable visual recovery. Tang et al. demonstrated that larger defects and more extensive outer retinal disruption were linked to unsatisfactory postoperative vision, while Wang et al. highlighted the prognostic value of ellipsoid zone impairment and macular hole configuration [6,7]. Collectively, these findings emphasize the role of detailed preoperative OCT analysis in estimating visual outcomes following TMH repair.

Interestingly, HH emerged as a significant factor associated with anatomical closure, whereas MLD and BD were not significantly related to closure outcomes in our cohort. Although larger horizontal dimensions are traditionally considered unfavorable prognostic indicators, the present findings suggest that vertical hole configuration may also contribute to closure dynamics in TMHs [9,14]. Increased HH may reflect preserved retinal elasticity or reduced chronic tissue loss, potentially facilitating approximation of the retinal edges during surgery. Given the limited research addressing the role of HH in TMHs, these findings warrant further validation in larger prospective studies.

The role of flap-based techniques in TMH surgery remains incompletely defined. In the present study, flap-assisted procedures achieved higher anatomical closure rates compared with non-flap techniques. Importantly, baseline OCT characteristics and preoperative BCVA were comparable between the groups, suggesting that the observed difference was unlikely to be solely attributable to baseline disease severity. Previous studies evaluating inverted or free ILM flap techniques in TMHs have reported variable results. While some authors found no clear superiority over conventional ILM peeling, others demonstrated favorable anatomical outcomes, particularly in large or chronic holes [9,13,15]. Our findings support the potential anatomical advantage of flap-assisted techniques in TMH repair.

This study has several limitations. First, its retrospective design may introduce selection bias. Second, the relatively limited sample size restricts statistical power. Third, surgical techniques and tamponade selection were determined at the surgeon’s discretion, which may have contributed to treatment heterogeneity. In addition, variability in trauma severity, associated ocular injuries, and the timing of surgical intervention may have influenced both anatomical and functional outcomes. Because associated traumatic findings, such as traumatic cataract, choroidal rupture, and vitreous hemorrhage, were heterogeneous and relatively infrequent, reliable subgroup analyses to determine their independent prognostic impact could not be performed. Finally, OCT evaluation was primarily based on structural parameters, and additional functional imaging biomarkers were not assessed.

Despite these limitations, the present study has several strengths, including detailed OCT-based morphological analysis, relatively long follow-up duration, and direct comparison of flap-based and non-flap surgical approaches in TMHs. Furthermore, the identification of HH as a potential factor associated with anatomical closure represents a potentially valuable observation that may contribute to future investigations in this field. Future prospective multicenter studies with larger cohorts are warranted to validate the prognostic role of hole height and to establish standardized OCT-based criteria for selecting the optimal surgical technique in traumatic macular holes.

## 5. Conclusions

In conclusion, PPV for TMH was associated with high anatomical closure rates and significant postoperative visual improvement. Larger macular hole dimensions were associated with poorer visual outcomes, whereas greater HH was associated with successful anatomical closure. Flap-based surgical techniques demonstrated higher anatomical success rates compared with conventional ILM peeling. These findings highlight the importance of preoperative OCT evaluation in predicting surgical outcomes and guiding management strategies in TMHs.

## Figures and Tables

**Table 1:** Baseline demographic and clinical characteristics of the study population.

Parameter	Value
Age (years)	28.7 ± 15.0
Sex (male/female)	31/2
Trauma type (blunt/penetrating)	29/4
Eye (right/left)	18/15
Lens status (phakic/pseudophakic)	31/2
Follow-up duration (months)	29.0 ± 27.2
Trauma-to-surgery interval (weeks)	10 (IQR, 4–24)
Associated ocular findings	22 (66.7%)
Choroidal rupture	8 (36.4%)
Berlin edema	7 (31.8%)
Mild vitreous hemorrhage	4 (18.2%)
Subretinal hemorrhage	3 (13.6%)

Data are presented as mean ± standard deviation, median (interquartile range), or number (%), as appropriate.

**Table 2:** Preoperative OCT characteristics and comparison between eyes with and without anatomical closure.

Parameter	Overall (*n* = 33)	No Closure (*n* = 5)	Closure (*n* = 28)	*p*-Value
Minimum linear diameter (µm)	597.6 ± 282.5	640.2 ± 296.9	588.1 ± 284.2	0.690
Basal diameter (µm)	1266.0 ± 459.2	1278.5 ± 558.0	1263.2 ± 446.7	0.943
Hole height (µm)	389.6 ± 130.1	265.7 ± 24.3	417.1 ± 128.0	**0.008**
Intraretinal cysts	21 (63.6%)	3 (60.0%)	18 (64.3%)	1.000
Subretinal fluid	12 (36.4%)	3 (60.0%)	9 (32.1%)	0.318
Epiretinal membrane	11 (33.3%)	2 (40.0%)	9 (32.1%)	1.000
Ellipsoid zone disruption	27 (81.8%)	4 (80.0%)	23 (82.1%)	1.000

Continuous variables were compared using the Mann–Whitney U-test. Categorical variables were compared using Fisher’s exact test.

**Table 3:** Comparison between flap-based and non-flap surgical techniques.

Parameter	Flap (*n* = 24)	Non-Flap (*n* = 9)	*p*-Value
Minimum linear diameter (µm)	608.2 ± 297.6	564.4 ± 243.9	0.709
Basal diameter (µm)	1238.0 ± 457.9	1353.6 ± 483.0	0.544
Hole height (µm)	404.0 ± 139.7	344.5 ± 85.7	0.266
Preoperative BCVA (logMAR)	1.28 ± 0.57	1.50 ± 0.41	0.330
Final BCVA (logMAR)	0.94 ± 0.67	0.96 ± 0.25	0.951
Age (years)	27.2 ± 14.4	33.5 ± 16.8	0.304
Anatomical closure	23/24 (95.8%)	5/9 (55.6%)	**0.013**

BCVA: Best-corrected visual acuity; logMAR: Logarithm of the minimum angle of resolution. Continuous variables were compared using the Mann–Whitney U-test. Categorical variables were compared using Fisher’s exact test.

## Data Availability

The datasets used and/or analyzed during the current study are available from the corresponding author on reasonable request.

## References

[1] Budoff G., Bhagat N., Zarbin M.A. (2019). Traumatic Macular Hole: Diagnosis, Natural History, and Management. J. Ophthalmol..

[2] Gao M., Liu K., Lin Q., Liu H. (2017). Management Modalities for Traumatic Macular Hole: A Systematic Review and Single-Arm Meta-Analysis. Curr. Eye Res..

[3] Bor’i A., Al-Aswad M.A., Saad A.A., Hamada D., Mahrous A. (2017). Pars Plana Vitrectomy with Internal Limiting Membrane Peeling in Traumatic Macular Hole: 14% Perfluoropropane (C3F8) versus Silicone Oil Tamponade. J. Ophthalmol..

[4] Zhou Q., Feng H., Lv H., Fu Z., Xue Y., Ye H. (2021). Vitrectomy vs. Spontaneous Closure for Traumatic Macular Hole: A Systematic Review and Meta-Analysis. Front. Med..

[5] Liu W., Grzybowski A. (2017). Current Management of Traumatic Macular Holes. J. Ophthalmol..

[6] Tang Y.F., Chang A., Campbell W.G., Connell P.P., Hunyor A.P., McAllister I.L., Essex R.W., Australian and New Zealand Society of Retinal Specialists (ANZSRS) Macular Hole Study Group (2020). SURGICAL MANAGEMENT OF TRAUMATIC MACULAR HOLE: Optical Coherence Tomography Features and Outcomes. Retina.

[7] Wang M., Yu Y., Wang Z., Liang X., Liu W. (2021). Surgical Treatment for Traumatic Macular Holes: Reconstructive Changes in Foveal Microstructures and Visual Predictors Analysis. Ophthalmologica.

[8] Arda H., Maier M., Schultheiß M., Haritoglou C. (2024). Advances in management strategies for large and persistent macular hole: An update. Surv. Ophthalmol..

[9] Cui Y., Wang G., Shi X. (2025). Characteristics and surgical outcomes of pediatric traumatic macular holes. BMC Ophthalmol..

[10] Kunikata H., Osada U., Abe T., Nakazawa T. (2021). Efficacy of early microincision vitrectomy surgery in traumatic macular hole. Graefe’s Arch. Clin. Exp. Ophthalmol..

[11] Venugopal R., Das A.V., Takkar B., Stewart M.W., Narayanan R. (2024). Real-world experience of full-thickness traumatic macular hole among young patients. Int. J. Retin. Vitr..

[12] Ruban A., Prudyus V., Zolnikova A., Petrovski B.É., Petrovski G., Binder S., Grzybowski A., Lytvynchuk L.M. (2026). Characteristics and surgical outcomes of combat blast-related full-thickness traumatic macular holes: Insights from war eye trauma in Ukraine. Int. J. Retin. Vitr..

[13] Ghoraba H.H., Leila M., Ghoraba H., Heikal M.A., Mansour H.O. (2019). Optical Coherence Tomography Morphological Features Following Modified Internal Limiting Membrane Surgical Technique In Traumatic Macular Holes. Clin. Ophthalmol..

[14] Kitic N., Chehaibou I., Chapron T., Metge F., Caputo G., Abdelmassih Y. (2025). Traumatic Macular Hole in Children: Characteristics, Management and Outcomes. Retina.

[15] Abou Shousha M.A. (2016). Inverted Internal Limiting Membrane Flap For Large Traumatic Macular Holes. Medicine.

